# Short-Chain Fatty Acids Production from Anaerobic Fermentation of Sewage Sludge: The Effect of Higher Levels Polyaluminium Chloride

**DOI:** 10.3390/ijerph19052806

**Published:** 2022-02-28

**Authors:** Puli Zhu, Xiaoyun Li, Jing Feng, Rui Zhang, Hui Bai, Duo Bu, Zeng Dan, Wei Li, Xuebin Lu

**Affiliations:** 1 School of Environmental Science and Engineering, Tianjin University, Tianjin 300072, China; zpl_tt@163.com (P.Z.); jingf@tju.edu.cn (J.F.); 2018214121@tju.edu.cn (H.B.); 2 School of Agriculture, Sun Yat-sen University, Guangzhou 510275, China; 3 School of Environmental and Municipal Engineering, Tianjin Chengjian University, Tianjin 300384, China; rzhang@tcu.edu.cn; 4 Department of Chemistry and Environmental Science, School of Science, Tibet University, Lhasa 850000, China; phudor@163.com (D.B.); yongzhong2008@163.com (Z.D.); li_wei05416@163.com (W.L.)

**Keywords:** polyaluminum chloride, sewage sludge, anaerobic fermentation, short-chain fatty acids

## Abstract

With the annual increase in the sludge production in China’s sewage treatment plants, the problem of sewage sludge treatment and disposal is becoming more and more serious. Anaerobic fermentation can convert complex organic matter in sewage sludge into short-chain fatty acid, hydrogen, methane and other resources and is an effective method for sewage sludge treatment and disposal. At the same time, sewage sludge often contains flocculants, which will inevitably affect the effect of anaerobic fermentation. As a high-performance flocculant, polyaluminum chloride (PAC) is widely used in wastewater treatment and sewage sludge dewatering processes. Previous studies indicated that lower levels of PAC inhibit the effect of the anaerobic fermentation process of sewage sludge; on the other hand, it is necessary to understand the effects of higher levels of PAC in anaerobically fermented sewage sludge. The results showed that higher levels (0.2–1 g Al/g total solids (TS)) of PAC could promote acid production from anaerobically fermented sewage sludge. Moreover, mechanism studies suggest that higher levels (0.2–1 g Al/g total solids (TS)) of PAC caused excessive adsorption of the charge on the surface of the sewage sludge colloid and reversed the charge. The sewage sludge colloid was stabilized again, which increases the concentration of soluble proteins, polysaccharides, and soluble extracellular polymers (S-EPS) in the fermentation broth, thereby improving the anaerobically fermented sewage sludge efficiency. The results provided from this study may act as technical reference and guidance for the engineering application of sewage sludge anaerobic fermentation.

## 1. Introduction

Biological wastewater treatment is widely used in the world because it is considered as an effective and inexpensive pollution treatment [1,2], but a significant amount of sewage sludge is produced during this process. It is estimated that the European Union annually produces more than 50 million tons of sewage sludge (calculated based on 80% moisture content) [3], while China produced 35 million tons of sewage sludge (calculated based on 80% moisture content) in 2015, and this number is expected to trend upward [4]. More and more sewage sludge poses challenges but also opportunities. The challenge lies in the complex composition of sewage sludge, which contains many pollutants, including pathogens, parasites, heavy metal elements, etc. If improperly handled and disposed of, it will pollute the environment and even endanger the safety of surrounding residents [5]. Opportunity lies in the sewage sludge as it also contains a large amount of biodegradable organic substrates [6]; if the resources are turned into bioenergy, waste can be turned into treasure [7].

In the context of the rapid increase in sewage sludge in urban sewage plants and the increasing shortage of energy in China, anaerobic fermentation has become an important method for the rapid stabilization and resource utilization of sewage sludge. While making full use of the organic matter in the sewage sludge, it can also produce short-chain fatty acids (SCFAs), hydrogen, methane, etc., which realizes sewage sludge reduction and resource utilization [8,9]. Among them, short-chain fatty acids have attracted much attention owing to their high added value [10].

However, sewage sludge often contains flocculants, which will inevitably affect the effect of anaerobic fermentation. Polyaluminium chloride (PAC), as a high-performance flocculant, is widely used in sewage treatment and sewage sludge dewatering processes, which makes PAC highly accumulate in sewage sludge [11,12]. PAC is a colorless or yellow resinous powder, which has the properties of adsorption, coagulation, and precipitation. PAC can destabilize the pollutants in the water and flocculate and aggregate into a larger volume of particulate matter, so that it can be easily separated from the water and achieve the purpose of purifying the water [13]. It is generally believed that Al_13_ is the best agglomerating and flocculating component of PAC, which is liable to undergo electrostatic neutralization and adsorption bridging effects with the active groups on the surface of sewage sludge particles, enabling fine particles to aggregate and be easily separated from water [14]. However, when the PAC is increased to a certain dose, the sewage sludge particles will be stabilized in the sewage sludge matrix again and no longer flocculate [13]. Chen et al. [15] studied the effect of polyaluminum chloride on anaerobically fermented sewage sludge, and the results showed that the presence of 30 mg Al/g TS of PAC in sewage sludge significantly reduces the production of short-chain fatty acids in sewage sludge anaerobic fermentation compared with the control group. The mechanism study shows that the inhibition phenomenon is caused by the flocculation of PAC. However, there was no relevant report on the effect of higher levels of PAC on anaerobically fermented sewage sludge. In addition, the levels of PAC have regional differences in sewage sludge, such as 81.6 mg Al/g TS in Hangzhou, 2.6–17.4 mg Al/g TS in Taiwan, 112 mg Al/g TS in India, and 320 mg Al/g TS in dewatered sewage sludge in the United States [15,16]. So, it is necessary to understand and master the effects and mechanisms of higher levels of PAC in sewage sludge on anaerobic fermentation.

Therefore, the aim of this work is to study the effect of higher levels of PAC on anaerobically fermented sewage sludge. The production of proteins, polysaccharides, and soluble chemical oxygen demand (SCOD) was examined by monitoring the degradation of organic matter. By monitoring the changes of sewage sludge flocs, the influence mechanism of PAC was proposed. In addition, cell membrane integrity was also analyzed. To our knowledge, this is the first time that the effect of higher levels of PAC on anaerobic fermentation of sewage sludge has been investigated. This provides technical reserves and theoretical guidance for the engineering application of the anaerobic fermentation of sewage sludge.

## 2. Materials and Methods

### 2.1. Sewage Sludge and Agent

The sewage sludge used in this study was collected from the secondary sedimentation tank of the sewage treatment plant in Tianjin, China, and no PAC pretreatment was allowed to eliminate the interference of the PAC background in the sludge. The raw sewage sludge was gravity-deposited in the laboratory for 24 h to remove the supernatant, and then the concentrated sewage sludge was stored in a refrigerator at 4 °C for anaerobic fermentation and domestication of inoculated sewage sludge. The main characteristics of the concentrated sewage sludge were as follows: pH 6.9 ± 0.1, total solids (TS) 25.1 ± 0.37 g/L, volatile solids (VS) 16.8 ± 0.21 g/L, total chemical oxygen demand (TCOD) 19.8 ± 0.45 g/L, SCOD 0.2 ± 0.01 g/L, total protein, 9.7 ± 0.33 g/L, total carbohydrate 2.2 ± 0.12 g/L.

In this study, Polyaluminum Chloride was analytical grade and provided by Tianjin Damao Chemical Reagent Factory. All the reagents used were analytical grade unless otherwise specified.

### 2.2. Experimental Design

According to the different dosages in which PAC may exist in sewage sludge, we designed the experiment as follows: Batch testing was performed in 12 replicate serum vials, first feeding with 400 mL of concentrated sewage sludge and 50 mL of inoculated sewage sludge in each vial. Different volumes of prepared PAC stock solution were added to each of the 11 reactors to achieve predetermined doses. The predetermined doses of PAC were added at 0.01, 0.05, 0.07, 0.1, 0.15, 0.2, 0.25, 0.3, 0.5, 0.7, and 1 g Al/g TS. The reactor without PAC was used as a control. All reactors were flushed with nitrogen gas for approximately 2 min to remove oxygen, and then the bottles were sealed with a rubber stopper and placed in an air bath shaker at a stirring speed of 120 rpm and a temperature of 37 °C for 7 days without pH control. During this period, all other anaerobic operating conditions were the same as described above unless otherwise stated.

### 2.3. Comparison of the Influence of PAC on Hydrolysis, Acidification, and Methanogenesis Processes Using the Degradation Efficiency of Model Substrates

The rates of hydrolysis, acidification, and methanogenesis can be expressed by the degradation rates of model compounds in synthetic wastewater; therefore, it is necessary to compare the effects of PAC on the processes of hydrolysis, acidification, and methanogenesis using these model substrates. Test-I compared the influences of various concentrations of PAC on the process of hydrolysis. Test-II and Test-III compared the effects of various concentrations of PAC on acidification and methanation, respectively [17].

Test-I: The main component of the synthetic wastewater is 3.0 g of bovine serum albumin (BSA average Mw 67,000, model protein)/L and 0.3 g of dextran (molecular weight (Mw) 23,800, model carbohydrate)/L. Four reactors were used for the simulation experiment. Each serum bottle received 400 mL of synthetic wastewater and 50 mL of inoculated sewage sludge, then 0, 0.1 and 0.3 g Al/g TS were added to each serum bottle. Other anaerobic fermentation operating conditions were the same as described in Section 2.2.

Test-II: BSA and dextran in the synthetic wastewater were replaced by 3 g L-alanine/L and 0.625 g glucose/L. The other components and experimental conditions were consistent with the hydrolysis process.

Test-III: The main component of the synthetic wastewater was sodium acetate (1.0 g/L), and the other components and experimental conditions did not change.

The degradation efficiency of the model compound can be calculated by the following equation:

Degradation efficiency (%) = 100 ∗ (C_0_ − Ct)/C_0_ (1)

where C_0_ represents the initial concentration of the model compound during the fermentation test, and Ct is the concentration of the model compound measured at a certain fermentation time.

### 2.4. EPS Extraction

Extraction of activated sewage sludge was conducted using the thermal extraction method [18]. A total of 50 mL of sewage sludge suspension was centrifuged (TGL16M, Changsha Xiang Yi centrifuge Co., Ltd., Changsha, China) at 4000× *g* for 5 min, and the supernatant was used for soluble extracellular polymer (S-EPS) measurement. The remaining sewage sludge particles in the tube were diluted with a 0.05% NaCl solution to the original volume of 50 mL, heated to 70 °C, then the sewage sludge suspension was sheared with a magnetic stirrer for 1 min and centrifuged at 4000× *g* for 10 min. The organic matter in the supernatant was measured to determine loosely bound EPS (L-EPS). The sewage sludge settled in the tube was resuspended in a 0.05% NaCl solution to a volume of 50 mL. The sewage sludge suspension was warmed to 60 °C in a water bath for 30 min, and the sewage sludge mixture was centrifuged at 4000× *g* for 15 min. The supernatant was collected and treated as a tightly bound EPS (T-EPS) of sewage sludge.

### 2.5. Analysis Method

The sewage sludge sample was centrifuged at 8000 rpm for 10 min and immediately filtered through 0.45 μm cellulose membrane. The supernatant obtained was used to determine soluble COD, SCFAs, soluble protein, and polysaccharides [19]. The BSA was used as a standard substance, and the soluble protein was measured using the Lowry method (Lowry Protein Assay Kit, Solarbio PC0030, Beijing Soleibo Technology Co., Ltd., Beijing, China) according to the manufacturer’s instructions. Polysaccharides were determined using the anthrone-sulfuric method using glucose as a standard substance [20]. SCOD was measured by dichromate titration, and TS and VS were determined as assayed by weighing method [21]. Cell membrane integrity was measured using a lactic dehydrogenase (LDH) Assay Kit (Solarbio BC0680, Beijing Soleibo Technology Co., Ltd., Beijing, China) according to the manufacturer’s instructions. Excitation emission matrix (EEM) fluorescence spectroscopy (Hitachi F4600, Tokyo, Japan) was used to characterize changes in extracellular polymers according to previously reported methods [22], measured in scan mode on a fluorospectrophotometer. Using a xenon lamp as the excitation light source, the EEM spectra of the samples were characterized by scanning the spectrum from 220 to 550 nm in 5 nm increments by changing the excitation Ex wavelength from 200 to 400 nm in 5 nm increments. The spectra were recorded at a scan rate of 12,000 nm/min using a 5 nm excitation and emission slit bandwidth. The voltage of the photomultiplier tube (PMT) was set to 700 V for low-level light detection [23]. SCFAs were determined using a high-performance liquid chromatography (HPLC, waters, e2695, Milford, CT, USA) unit with ultraviolet (UV) detection (λ = 210 nm) equipped with a C_18_ chromatographic column. The injection volume was 10 μL, and the mobile phases used were phosphate buffered saline (10 mmol/L, pH = 2.5) and methanol (78:22, *v/v*) circulated at 1.0 mL/min at 30 °C [24].

## 3. Results and Discussion

### 3.1. Effect of Polyaluminum Chloride on Short-Chain Fatty Acids Produced by Anaerobically Fermented Sewage Sludge

**Figure 1:**
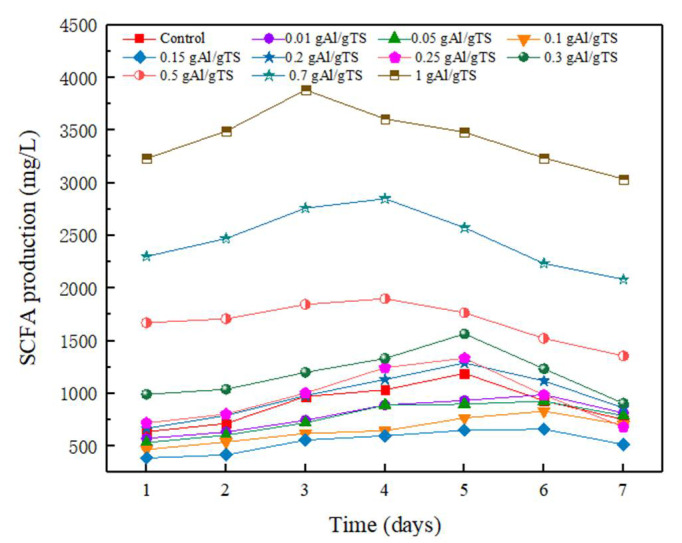
SCFAs production from anaerobically fermented sewage sludge with different PAC level.

SCFAs production is an important parameter in assessing the anaerobic fermentation efficiency of sewage sludge [25]. Figure 1 shows the curves for the production of SCFAs in anaerobic fermentation at different PAC levels. It can be seen that as the PAC increases from 0–0.15 g Al/g TS, the maximum SCFAs yield is reduced from 1190 mg/L to 659 mg/L and the production of SCFAs is inhibited, which agrees with the previous literature [15]. However, further increases in PAC lead to greater SCFAs accumulation. Particularly, when the PAC concentration was 1 g Al/g TS, the maximum SCFAs production was 3880 mg/L, which is 3 times that of the control and 6 times greater than that of a PAC concentration that was at 0.15 g Al/g TS. The results showed that the effect of PAC on SCFAs production during the anaerobic fermentation of sewage sludge was dose-dependent. Lower levels of PAC (<0.15 g Al/g TS) inhibited the production of SCFAs, while higher levels of PAC (>0.2 g Al/g TS) promoted the accumulation of SCFAs. SCFAs production increased gradually as fermentation time increased, while further increases in fermentation time caused a decrease in SCFAs production in all fermenters. The decrease in SCFAs production was attributed to their consumption during methane generation.

### 3.2. Analysis of Substrate Degradation

With the aim to explore the reasons and mechanisms that affect the different acid production, this section analyzes the source of the acid production reaction: substrate degradation rate. In the process of anaerobic fermentation, the acid-producing bacteria mainly take soluble proteins and carbohydrates in sewage sludge mixture as fermentation substrates to metabolize and produce short-chain fatty acids. The organic matter in the liquid phase primarily comes from the dissolution of the substances in the sewage sludge cells or the release of organic matter from the EPS.

#### 3.2.1. Effect of Polyaluminum Chloride on Dissolution of Soluble Substrates

**Figure 2:**
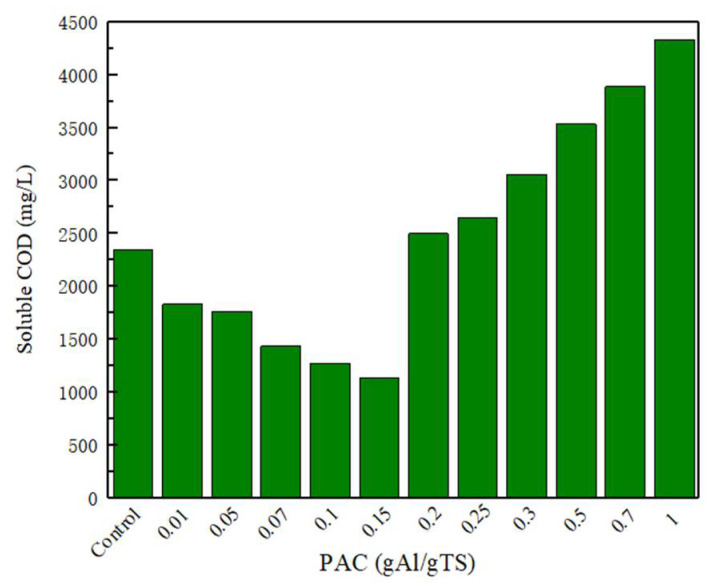
SCOD concentration with different PAC level.

Sewage sludge dissolution is considered as the most critical step in the anaerobically fermented sewage sludge. The dissolved organic matter can provide an available substrate for microbial fermentation, and the acid production efficiency of anaerobically fermented sewage sludge could be improved [26]. The soluble proteins, soluble polysaccharides, and SCOD have obvious impacts on the dissolution efficiency of sewage sludge [27]. Figure 2 shows the changes in SCOD in the sewage sludge fermentation broth at different concentrations of polyaluminum chloride. As shown in Figure 2, as the concentration of polyaluminum chloride changed from 0 to 0.15 g Al/g TS, the SCOD concentration decreased from 2350 mg/L to 1140 mg/L. However, as the polyaluminum chloride further increased, the concentration of SCOD significantly improved. The results showed that the higher levels of PAC (>0.2 g Al/g TS) promoted the dissolution of SCOD. 

**Figure 3:**
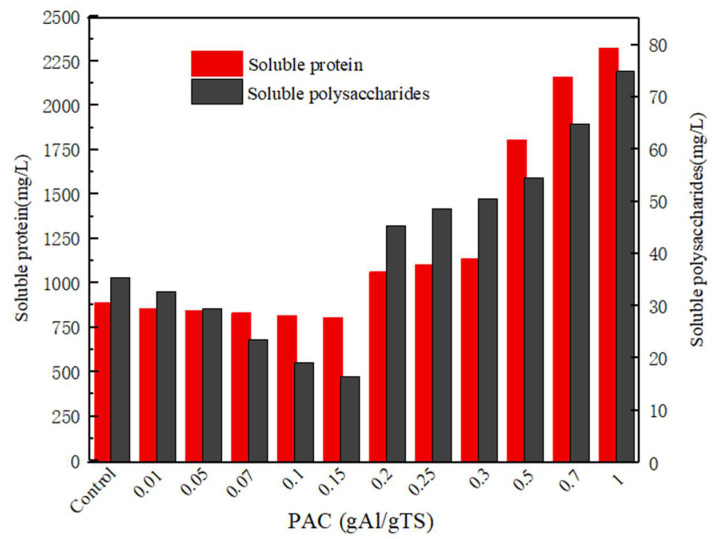
Soluble protein and soluble carbohydrate concentration with different PAC level.

It can be observed in Figure 3 that the soluble protein and polysaccharide have similar changes under different concentrations of polyaluminum chloride. When the concentration of PAC changes from 0.2 to 1 g Al/g TS, the concentration of soluble protein and polysaccharide also increase accordingly. The results showed that the higher levels of PAC (>0.2 g Al/g TS) promoted the dissolution of soluble protein and polysaccharide. The phenomenon indicated that high concentrations of PAC was beneficial for the dissolution of more soluble organic matter. The result is consistent with the effect of polyaluminum chloride on the accumulation of short-chain fatty acids during anaerobically fermented sewage sludge.

#### 3.2.2. Effect of Polyaluminum Chloride on EPS Dissolution in Sludge

EPS accounts for about 80% of the mass of activated sewage sludge. It is an organic polymer secreted by microorganisms in activated sewage sludge and attached to the microbial cell wall [28]. It mainly consists of proteins and carbohydrates produced by microorganisms, which could protect cells from harmful environmental effects. Therefore, the EPS affected the rate of sewage sludge dissolution [29]. Generally, EPS can be divided into three layers, S-EPS, L-EPS, and T-EPS from the inside to the outside. Changes in the contents of each layer can reflect the sewage sludge cracking process [23]. Most of the dissolved organic matter in the sewage sludge contains the conjugated double-bond aromatic hydrocarbons or conjugated systems such as double bonds, carbon groups, and carboxyl groups, which emit fluorescences of different wavelengths under the excitation and irradiation of specific wavelength light in the ultraviolet region [7]. The three-dimensional fluorescence method represents the fluorescence intensity as a function of two variables of excitation wavelength and emission wavelength, that is, a three-dimensional fluorescence spectrum. The spectrum can demonstrate the fluorescence intensity information when the excitation wavelength (λex) and emission wavelength (λem) are changed simultaneously, which can reveal the organic classification of pollutants and their content information [23]. It has been reported that fluorescence peak position shift can be used to characterize changes in EPS structure, while fluorescence intensity can be applied to show the alterations of EPS content. Based on this principle, a three-dimensional fluorescence spectrum can be utilized to determine the soluble organic matter in the sewage sludge.

**Figure 4:**
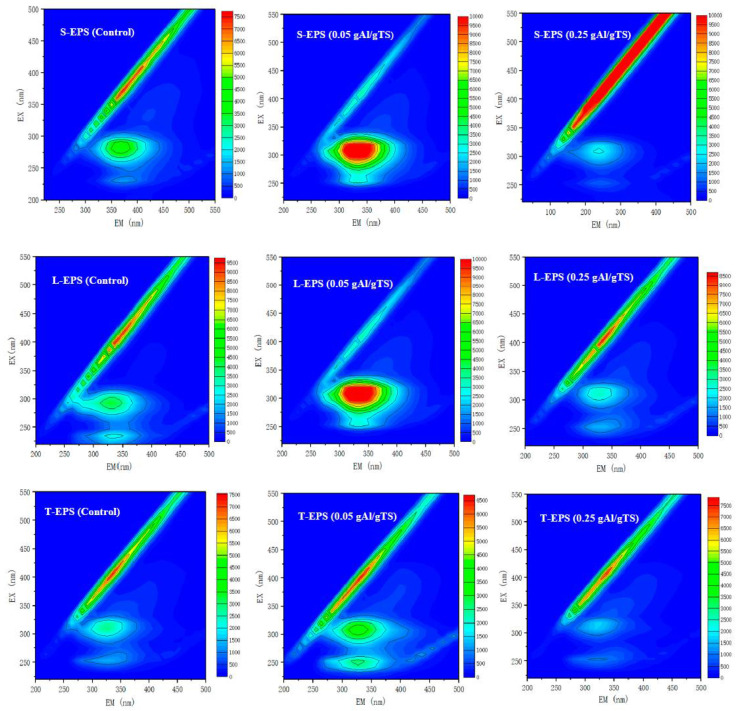
EEM spectra of different EPS fractions (S-EPS, L-EPS, T-EPS) with different PAC levels.

In this study, S-EPS, L-EPS, and T-EPS were extracted from sewage sludge with 0, 0.05, and 0.25 g Al/g TS, respectively. The EEM fluorescence spectra are shown in Figure 4. The characterization of EPS by EEM spectroscopy indicated that two main peaks were found in sewage sludge. The peak at the excitation/emission wavelength (Ex/EM) 225/335 nm is assigned to an aromatic proteinaceous substance. The Ex/EM is identified as a tryptophan protein-like substance at approximately 280/335 nm [21]. Compared with the control (0 g Al/g TS), the addition of 0.05 g Al/g TS caused a redshift in the fluorescent peak and an increase in the fluorescence intensity. The results suggest that the PAC with 0.05 g Al/g TS sewage sludge contained more T-EPS than the PAC with 0 g Al/g TS sewage sludge, which resulted in a stronger defense ability for the sewage sludge cells against PAC, preventing the release of intracellular substances. Meanwhile, 0.25 g AL/g TS contains relatively little T-EPS, which contributes to the release of large amounts of extracellular and intracellular material from the sewage sludge.

It is generally believed that the level of soluble substrate in the sewage sludge fermentation system will increase with the concentration of toxic compounds [24]. Aluminum ions are one of the heavy metal ions that affect biological systems, causing oxidative damage to cell membranes and leading to the leakage of intracellular substrates [30]. Therefore, LDH release assays were used to study the possible toxicity mechanism of PAC in this study [31]. The results showed that the presence of PAC did not cause additional leakage of intracellular substrate. Thus, the release of soluble substances in the sewage sludge could be attributed to the secretion of EPS.

### 3.3. Comparison of the Influence of Various PAC Levels on Hydrolysis, Acidification, and Methanogenesis Processes

After the sewage sludge is dissolved, the dissolved substrate will undergo hydrolysis, acidogenic, and methanogenic processes. To further investigate the influence of PAC addition on hydrolysis, acidification, and methanation, the degradation rate of model compounds in synthetic wastewater was explored. Table 1 shows the effect of PAC on the degradation of simulated compounds during hydrolysis, acidification, and methanogenesis. The degradation rates of BSA and glucan treated at a concentration of 0.1 g Al/g TS were 4.32% and 7.89% on the third day, respectively. However, when the level of PAC was 0.3 g Al/g TS, the degradation rate of BSA and dextran at 3 days increased to 19.2% and 96.3%, which was significantly higher than 0.1 g Al/g TS. The above experimental data showed that higher levels of PAC (>0.2 g Al/g TS) have a positive effect on the sewage sludge hydrolysis process. The level of PAC on acidification was similarly studied. Degradation efficiencies of L-alanine over 3 days in the presence of various levels of PAC (0, 0.1 and 0.3 g Al/g TS) were 22.6%, 4.96%, and 27.1%, respectively. For glucose, when PAC was added to the fermentation system, the acidification efficiency was similar, which showed that higher levels of PAC promote the degradation of L-alanine and glucose during sewage sludge acidification. The presence of PAC during methanogenesis significantly reduced the rate of degradation of acetate. For example, in a reactor without PAC, the degradation rate of acetate at 3 days was 24.6%, and the degradation rates of acetate in low- and high-level PAC reactors were 7.31% and 9.25%, respectively, which were much lower than the blank. The results indicated that higher levels of PAC (>0.2 g Al/g TS) promote the hydrolysis and acidification processes of sewage sludge. It is worth noting that differences in the rate of degradation of acetate by any level of PAC are not significant, the accumulation of SCFAs causes the pH to decrease. However, the acidic conditions exhibit a strong inhibitory effect on the activity of methanogens and inhibit methanogenesis [32].

### 3.4. Effect of Polyaluminum Chloride on the Flocculation Structure of Sewage Sludge in Anaerobic Fermentation

#### 3.4.1. Effect of Polyaluminum Chloride on Microstructure of Sewage Sludge

**Figure 5:**
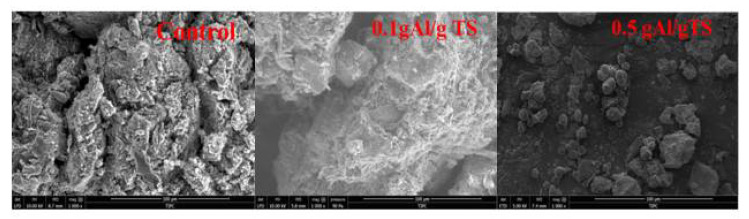
The microstructure of sewage sludge with different PAC level.

Figure 5 shows the micromorphology of sewage sludge flocs at various levels of PAC. As shown, when the PAC concentration was 0.1 g Al/g TS, the size of the sewage sludge particles are large and the sewage sludge flocs are compact and dense. These features could make the organic matter in the sewage sludge particles difficult to dissolve. This is because the PAC hydrolysis produces Alb, which carries a large positive charge, which can neutralize the negative charges on the surfaces of the sewage sludge particles. The suspension will be unstable, resulting in flocculation into dense sewage sludge flocs. However, when the PAC level was raised from 0.1 to 0.5 g Al/g TS, the sewage sludge particles became smaller and more dispersed. This is because the suspended particles of the sewage sludge were excessively adsorbed by the PAC. This behavior could cause the charge on the surface of the colloidal particles to reverse, then the suspended particles of the sewage sludge restabilized from the coagulation system, which helps to dissolve more soluble organic matter.

#### 3.4.2. Effect of EPS Change on Sewage Sludge Flocs Stability

EPS is an important component of sewage sludge floc. It plays an important role in the mass transfer performance, flocculation performance, sedimentation performance, dehydration performance, stability performance, metal ion adsorption performance, and granulation performance of sewage sludge. Furthermore, the EPS is helpful to maintain the structure of microbial aggregates and control the stability of the sewage sludge flocs [33]. There are many charged groups on the surface of EPS, which can enhance flocculation or prevent flocculation [34]. The repulsive force between sewage sludge cells is mainly affected by L-EPS, which plays a decisive role in the flocculation type of sewage sludge. Increasing the content of L-EPS will damage the cell attachment and deteriorate the flocculent structure [35]. EPS affects the sedimentation performance of sewage sludge by altering the physical and chemical properties of the sewage sludge flocs, such as hydrophilicity and hydrophobicity. The sedimentation performance of sewage sludge is mainly determined by T-EPS. Lower levels of T-EPS resulted in reduced sewage sludge stability and could expose more microorganisms to available organic matter [36].

**Figure 6:**
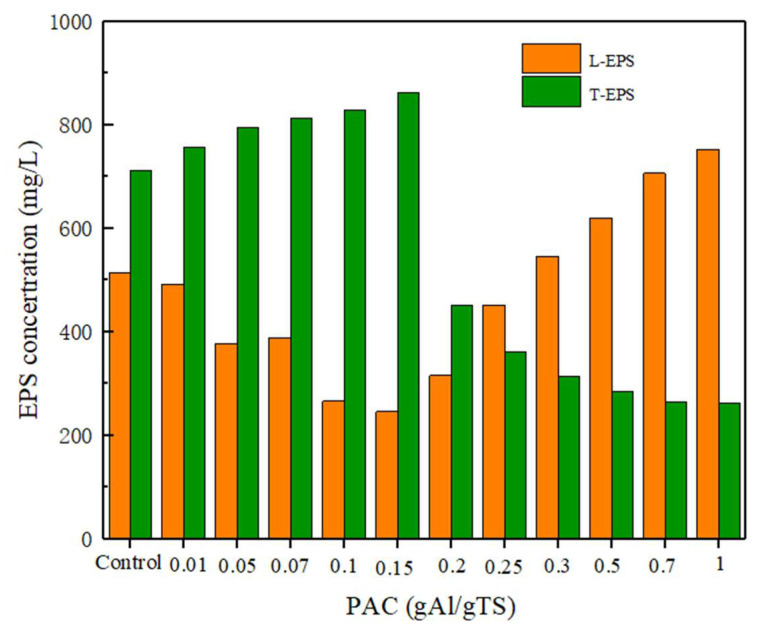
Effects of different level of PAC on sewage sludge EPS distribution.

In this study, the stability of sewage sludge flocs after PAC addition was evaluated by investigating changes in the composition and content of EPS. As shown in Figure 6, as the amount of PAC added increased from 0 to 0.15 g Al/g TS, the T-EPS content increased while the L-EPS content showed a decreasing trend. However, with the further increases in PAC, the T-EPS content decreased while L-EPS showed an increasing trend. The above results showed that higher levels of PAC (>0.2 g Al/g TS) impaired the stability of sewage sludge flocs, which illustrated that EPS diffused from the T-EPS of the sewage sludge flocs to the L-EPS. As a dispersible component, L-EPS and S-EPS are more easily dispersed or hydrolyzed by extracellular enzymes, which will further enhance the production of short-chain fatty acids [35,37]. This also confirms that the high level of polyaluminum chloride has a great influence on the distribution of EPS and the stability of sewage sludge flocs, which can promote the anaerobically fermented sewage sludge process.

## 4. Conclusions

In this work, the effect of higher levels of polyaluminum chloride on the production of short-chain fatty acids by anaerobic fermentation of waste-activated sewage sludge was investigated. The production of proteins, polysaccharides, and SCOD were evaluated by monitoring the degradation of organic matter. The mechanism of PAC was proposed by monitoring the changes of sewage sludge flocs. In addition, the integrity of the cell membrane was also analyzed. The results showed that the effect of PAC on SCFAs production during the anaerobic fermentation of sewage sludge was dose-dependent. Specifically, lower levels of PAC inhibited the production of SCFAs, while higher levels of PAC (>0.2 g Al/g TS) promoted the accumulation of SCFAs. The mechanism proposed in this study indicated that higher levels of PAC (>0.2 g Al/g TS) cause a reversal of the sewage sludge surface charge, causing the sewage sludge flocs to restabilize and become loose, thereby increasing the content of soluble and loosely bound extracellular polymers and promoting the dissolution and availability of more proteins, polysaccharides, and SCOD for microbial utilization. Further research showed that PAC did not cause damage to the sewage sludge cell wall, and the main dissolved substances were extracellular polymers. This work reveals the underlying mechanism of how higher levels of PAC affect the production of SCFAs during fermentation and will provide technical reserves and theoretical guidance for the engineering application of sewage sludge anaerobic fermentation in areas with higher PAC levels.

## Figures and Tables

**Table 1:** Effect of different level of PAC on the degradations of model compounds.

Item	Time	Hydrolysis		Acidogenesis		Methanogenesis
		BSA Degradation Equation (1) (%)	Detran Degradation Equation (1) (%)	L-Alanine Degradation Equation (1) (%)	Glucose Degradation Equation (1) (%)	Acetate Degradation Equation (1) (%)
0 g Al/g TS	1 d	9.55	42.3	15.4	45.6	12.3
	3 d	15.1	84.2	22.6	89.9	24.6
0.1 g Al/gTS	1 d	3.33	3.9	4.1	7.6	6.6
	3 d	4.32	7.89	4.96	68.6	7.3
0.3 g Al/gTS	1 d	15.9	51.9	21.9	56.5	7.3
	3 d	19.2	96.3	27.1	98.7	9.3
